# Dietary Polyphenols and Their Effects on Cell Biochemistry and Pathophysiology 2014

**DOI:** 10.1155/2015/782424

**Published:** 2015-07-21

**Authors:** Cristina Angeloni, Tullia Maraldi, Dragan Milenkovic, David Vauzour

**Affiliations:** ^1^Department for Life Quality Studies, Alma Mater Studiorum University of Bologna, Corso d'Augusto 237, 47921 Rimini, Italy; ^2^Department of Surgical, Medical, Dental and Morphological Sciences with Interest in Transplant, Oncology and Regenerative Medicine, University of Modena and Reggio Emilia, Via Del Pozzo 71, 41100 Modena, Italy; ^3^INRA, UMR 1019, UNH, CRNH Auvergne and Unité de Nutrition Humaine, Université d'Auvergne, Clermont Université, BP 10448, 63000 Clermont-Ferrand, France; ^4^Norwich Medical School, University of East Anglia, Norwich NR4 7UQ, UK

Epidemiological studies suggest that high dietary intake of phytochemicals and in particular of polyphenols is associated with decreased risk of a multitude of diseases states including cardiovascular disease, cancer, and neurodegenerative diseases.

As oxidative stress is involved in all these pathological conditions, the antioxidant properties of polyphenols and other natural compounds have attracted the interest of many authors.

M. Horvathova et al. investigated the effects of the intake of Robuvit, a water extract obtained from the wood of* Quercus robur*, showing that it is associated with decrease of markers of oxidative stress and increase of activity of antioxidant enzymes and total antioxidant capacity of plasma. As reactive oxygen species (ROS) are strongly associated also with skin aging, the study of K. Watanabe et al. investigated the antiatrophic effects of melinjo seed extract (MSE), containing transresveratrol and resveratrol derivatives, on age-related skin pathologies in Sod1^−/−^ mice. Their results demonstrated that MSE and transresveratrol significantly reversed skin thinning via reduction of oxidative damage.

Even if many of the positive biological actions of flavonoids have been assigned to their antioxidant properties, there is an emerging view that they might play other protective activities such as acting as anti-inflammatory agents. The study by O. Farkas et al. strengthened this point of view demonstrating that the flavonoids apigenin and its trimethylated analogue attenuated LPS-induced inflammation in IPEC-J2 nontransformed intestinal epithelial cells. The anti-inflammatory, antioxidative, and cytoprotective effects of resveratrol, epigallocatechingallate, genistein, apigenin, (−)-epicatechin, and other polyphenols have been reviewed by A. Malhotra et al., J. Shay et al., and S. Upadhyay and M. Dixit.

As far as the lipid metabolism is concerned, I. Eseberri et al. investigated the effect of doses of quercetin on triacylglycerol accumulation in maturing preadipocytes and mature adipocytes. They concluded that quercetin, in the range of serum concentrations, is able to inhibit adipogenesis, but higher doses are needed to reduce fat accumulation in mature adipocytes.

Regarding glucose metabolism, D. M. P. H. J. Boesten et al. examined the protective role of three structurally related flavonoids (rutin, quercetin, and flavone) during high glucose conditions in human umbilical vein endothelial cells. They conclude that this protective effect of flavonoids is a combination of the flavonoids abilities to inhibit both PARP activation and aldose reductase enzyme activity. The review by L. Basheer and Z. Kerem focuses on interactions between dietary polyphenols and CYP3A4.

Among the fruits presenting chemopreventive and/or chemotherapeutic potential is pomegranate that has been shown to exert anticancer activity which is generally attributed to its high content of polyphenols. E. Turrini et al. reviewed pomegranate antiproliferative, anti-invasive, and antimetastatic effects focusing on apoptosis induction and the inhibition of inflammatory pathways.

Polyphenolic extracts from the edible part of artichoke (*Cynara scolymus* L.) have also been shown to be potential chemopreventive and anticancer dietary compounds as they induce apoptosis and decrease the invasive potential of the human breast cancer cells (A. M. Mileo et al.).

Among the polyphenols present in fruits are proanthocyanidins that are the most widely represented products of plants secondary metabolism. C. Minker et al. showed that they reach the colon practically intact, where they are able to locally exert their anticancer activities on colorectal precancerous and cancerous cells and can exert proapoptotic activities. Together with proanthocyanidins, it has been observed that other families of polyphenols can exert variety of biological properties, including both antioxidant and nonantioxidant functions, as described in the review article by J. Shay et al. Finally, J. Wang et al. review epidemiological studies or controlled clinical trials which employed biomarkers of exposure for polyphenols to help assess their anticarcinogenic role and concluded that more evidences should be collected before a conclusion can be made towards their protective roles.

Atherosclerosis is the primary cause of cardiovascular diseases and high levels of LDL cholesterol (LDL-C) and LDL oxidation have been regarded as two important risk factors for atherogenesis. H. Han et al. designed a study to evaluate the effects of flaxseed oil containing *α*-linolenic acid ester of plant sterols (ALA-PS) on apoE knock-out mice that received high fat diet alone or supplemented with flaxseed oil with or without ALA-PS for 18 weeks. Results demonstrated that flaxseed oil containing ALA-PS improved overall lipid levels, inhibited inflammation, and reduced oxidative stress. M. A. Rahman et al. demonstrated that the polyphenolic compounds present in the* Flammulina velutipes*, also known as golden needle mushroom, inhibited LDL oxidation and they ascribed this chain-breaking activity to protocatechuic acid (PCA), p-coumaric acid, and ellagic acid. The study by R. Varì et al. expanded this knowledge trying to define the molecular mechanism responsible for the protective effects of PCA against oxidative and proapoptotic damage exerted by oxLDL in J774 A.1 macrophages. They demonstrated the essential role of JNK/Nrf2 signalling pathway in the antiapoptotic activity exerted by PCA in oxidatively stressed macrophages by improving the endogenous cellular antioxidant system.

As hypertension is another important risk factor for cardiovascular disease, M. Micucci et al. focused their attention on the potential application of* Olea europaea* L. leaf extract (OEE) and of a* Hibiscus sabdariffa* L. flower extract (HSE) in the prevention/counteraction of hypertension, a pathological condition affecting a large number of populations. They showed the antioxidant and cytoprotective effects of OEE and HSE and their ability to modulate cardiac inotropy and chronotropy together with their relaxant activity on vascular smooth muscle.

In a review paper, A. N. Orekhov et al. addressed the important issue of identifying natural compound as an alternative strategy for atherosclerosis prevention as most of the synthetic drugs available have severe side effects and high cost of treatment. Moreover, they analyzed the achievements in the development and use of models based on primary cultures of human aortic cells to test the antiatherosclerotic activity of compounds of natural origin.

Neurodegenerative diseases are dramatically increasing worldwide and this is strictly correlated to the aging of the world population; therefore, natural compounds could be an effective and cheap preventive strategy to maintain cognitive health with age.

F. Jagla and O. Pechanova reviewed the potential protective effects of polyphenols on the development of neurodegenerative diseases, in particular, focusing on endothelial dysfunction implicated in cognitive decline. The review by E. Tellone et al. explored the neuroprotective role of resveratrol in counteracting different neurodegenerative disorders. M. Reinisalo et al. analyzed resveratrol positive effects on the most common aging-related diseases clarifying the molecular mechanisms involved in the stilbene-mediated protection against oxidative stress. Carqueja (*Baccharis trimera*) is a native plant found throughout South America and its hydroalcoholic extract (CHE) has been demonstrated to have a protective effect in a* C. elegans* model of Alzheimer's disease (F. A. Paiva et al.). A. Masci et al. showed that a raw broccoli sprout juice protects neuroblastoma cells against A*β*-induced cytotoxicity via the induction of antiapoptotic signals, such as increased Hsp70 mRNA levels, and the activation of Nrf2-ARE signalling pathway upregulation of Nrf2-dependent antioxidant capacity.

Besides neurodegenerative disease and ageing, there are other disorders related to brain damage such as psychiatric disorders including major depression, attention deficit hyperactivity disease (ADHD) and schizophrenia, and cognitive deficit and brain damage induced by chronic alcohol consumption. N. Phunchago et al. studied the effects of* Tiliacora triandra* extract on memory impairment, neuron density, cholinergic function, and oxidative stress in hippocampus of alcoholic rats demonstrating that the extract improved memory deficit partly via decreasing oxidative stress and the suppressing AChE. Polyphenolic compounds can be also involved in modulation of mental health including brain plasticity, behavior, mood, depression, and cognition. J. Trebatická and Z. Duracková analyzed the large number of studies on the effects of natural polyphenols on mental disorders, and they concluded that even if polyphenols in the diet have the potential to become medicaments in the field of mental health, the use in clinical practice is still a long way off.

In conclusion, this special issue provides new findings on the role that polyphenols and other natural compounds play in the prevention of chronic degenerative diseases and expands our knowledge on the new mechanisms of actions of these pleiotropic compounds.

*Cristina Angeloni* *Cristina Angeloni* *Tullia Maraldi* *Tullia Maraldi* *Dragan Milenkovic* *Dragan Milenkovic* *David Vauzour* *David Vauzour*

